# Non-Immunogenicity of Overlapping Gag Peptides Pulsed on Autologous Cells after Vaccination of HIV Infected Individuals

**DOI:** 10.1371/journal.pone.0074389

**Published:** 2013-10-04

**Authors:** Henrik N. Kløverpris, Akil Jackson, Amanda Handley, Peter Hayes, Jill Gilmour, Lynn Riddell, Fabian Chen, Mark Atkins, Marta Boffito, Bruce D. Walker, Jim Ackland, Mark Sullivan, Philip Goulder

**Affiliations:** 1 Department of Paediatrics, University of Oxford, Oxford, United Kingdom; 2 St Stephen's AIDS Trust St Stephen's Centre, Chelsea and Westminster Hospital, London, United Kingdom; 3 Medicines Development, Melbourne, Victoria, Australia; 4 IAVI Human Immunology Laboratory, Imperial College, London, United Kingdom; 5 Department of Genitourinary Medicine, Northhamptonshire Healthcare National Health Service Trust, Northhampton General Hospital, Cliftonville, Northhampton, United Kingdom; 6 Department of Sexual Health, Royal Berkshire Hospital, Reading, United Kingdom; 7 KwaZulu-Natal Research Institute for Tuberculosis and HIV (K-RITH), Nelson R Mandela School of Medicine, University of Kwazulu-Natal, Durban, KwaZulu-Natal, South Africa; 8 Department of International Health, Immunology and Microbiology, University of Copenhagen, Copenhagen, Denmark; 9 Ragon Institute of Massachusetts General Hospital, Massachusetts Institute of Technology and Harvard, Boston, Massachusetts, United States of America; 10 Howard Hughes Medical Institute, Maryland, Chevy Chase, Maryland, United States of America; 11 Global Biosolutions, Craigeburn, Victoria, Australia; Rush University, United States of America

## Abstract

**Background:**

HIV Gag-specific CD4+ and CD8+ T-cell responses are important for HIV immune control. Pulsing overlapping Gag peptides on autologous lymphocytes (OPAL) has proven immunogenic and effective in reducing viral loads in multiple pigtail macaque studies, warranting clinical evaluation.

**Methodology:**

We performed a phase I, single centre, placebo-controlled, double-blinded and dose-escalating study to evaluate the safety and preliminary immunogenicity of a novel therapeutic vaccine approach ‘OPAL-HIV-Gag(c)’. This vaccine is comprised of 120 15mer peptides, overlapping by 11 amino acids, spanning the HIV Gag C clade sequence proteome, pulsed on white blood cells enriched from whole blood using a closed system, followed by intravenous reinfusion. Patients with undetectable HIV viral loads (<50 copies/ml plasma) on HAART received four administrations at week 0, 4, 8 and 12, and were followed up for 12 weeks post-treatment. Twenty-three people were enrolled in four groups: 12 mg (n = 6), 24 mg (n = 7), 48 mg (n = 2) or matching placebo (n = 8) with 18 immunologically evaluable. T-cell immunogenicity was assessed by IFNγ ELIspot and intracellular cytokine staining (ICS).

**Results:**

The OPAL-HIV-Gag(c) peptides were antigenic *in vitro* in 17/17 subjects. After vaccination with OPAL-HIV-Gag(c), 1/6 subjects at 12 mg and 1/6 subjects at 24 mg dose groups had a 2- and 3-fold increase in ELIspot magnitudes from baseline, respectively, of Gag-specific CD8+ T-cells at week 14, compared to 0/6 subjects in the placebo group. No Gag-specific CD4+ T-cell responses or overall change in Rev, Nef, Tat and CMV specific responses were detected. Marked, transient and self-limiting lymphopenia was observed immediately post-vaccination (4 hours) in OPAL-HIV-Gag(c) but not in placebo recipients, with median fall from 1.72 to 0.67 million lymphocytes/mL for active groups (P<0.001), compared to post-placebo from 1.70 to 1.56 lymphocytes/ml (P = 0.16).

**Conclusion/Significance:**

Despite strong immunogenicity observed in several *Macaca nemestrina* studies using this approach, OPAL-HIV-Gag(c) was not significantly immunogenic in humans and improved methods of generating high-frequency Gag-specific T-cell responses are required.

**Name of Registry:**

ClinicalTrials.gov, Registry number: NCT01123915, URL trial registry database: http://www.clinicaltrials.gov/ct2/results?term=OPAL-HIV-1001&Search=Search

## Introduction

A therapeutic HIV vaccine would add both a novel class of treatment and a potential alternative to life-long pharmaceutical therapy. However, despite approximately 3 decades of research, the goal of prophylactic and therapeutic HIV vaccines remains unfulfilled. The primary objective of a therapeutic vaccination is to induce (or boost pre-existent) antiviral T-cell responses to improve control of infection. HIV-specific CD8+ T-cell responses are critical for the control of virus replication during acute [1] and chronic infection [2], irrespective of the restricting HLA allele. Gag-specific CD8+ T-cell responses provide a major contribution to viral control [3], [4], [5] by direct activity against virally infected cells [6], [7], [8], [9].

A number of strategies have been employed to elicit such a desired immune response. Naked DNA vaccines have shown limited immunogenicity [10], [11], [12], [13] and adenoviral vectors that have been immunogenic [14] have been hampered by pre-existing vector-specific immunity [15], [16]. Second generation vaccines using chimpanzee or rare human adenovirus-based vectors, or cytomegalovirus vectors have shown promising results in non-human primates and humans [17], [18], [19], [20]. Delivery of peptides on the surface of professional antigen presenting cells, such as dendritic cells, circumvents the problems of vector-specific immunity [21] and has shown induction of both CD4+ and CD8+ T-cell responses [22], [23], [24], [25], [26]. However, generation of dendritic cells *ex vivo* for human vaccination is labour-intensive, costly, and requires specialised laboratory facilities for *in vivo* administration [27], [28]. This precludes broad dissemination of this treatment modality in most areas with high HIV seroprevalence, such as Sub-Saharan Africa.

OPAL (pulsing Overlapping Peptides on Autologous Lymphocytes) is a novel approach that has generated high-frequency and boostable, polyfunctional CD4+ and CD8+ T-cell responses in non-human primates [29], [30], [31]. In particular, re-infusion of fresh autologous PBMCs pulsed with overlapping SIV Gag peptides in SIV-infected macaques resulted in a 10-fold reduction of viral load set point after discontinuation of antiretroviral therapy (ART) sustained for 6 months. The peptides used can be manufactured to span all epitopes within the protein of interest and prior knowledge of the particular MHC class I molecules expressed is not required.

Here, we present the immunogenicity data from the first-in-human administration of OPAL-HIV-Gag(c).

## Methods

### Study design

The protocol for this trial and supporting CONSORT checklist are available as supporting information; see Checklist S1 and Protocol S1. This was a phase I, single centre, placebo-controlled, double-blind, dose-escalating study of the safety and preliminary immunogenicity of OPAL-HIV-Gag(c) in HIV positive adults receiving stable ART.

### Ethics statement

The OPAL-HIV-1001 study was conducted at a single site in the United Kingdom under the Medicines and Healthcare products Regulatory Agency (MHRA) Clinical Trials Authorisation (CTA) scheme. The EudraCT number for the study was 2008-005142-23. Receipt of acknowledgment from the MHRA for the study was obtained prior to study commencement on 26 Feb 2010. Approval for conduct of the study was obtained from the Independent Ethics Committee (IEC), The Royal Marsden Research Ethics Committee, St Georges University of London, Blackshaw Road, Tooting, London SW17 0RE, United Kingdom, associated with the study site before study commencement. In addition to the approval for the conduct of the study from the IEC associated with the site, the protocol was submitted to the local Institutional Review Board (IRB) of Partners Human Research Committee, Massachusetts General Hospital, Boston, Massachusetts, United States of America. Expedited approval and notification of the determinations of the MHRA and IEC were sufficient without the requirement for full review by this IRB. The IRB also received all amendments to the protocol, annual reports, the Investigators Brochure (IB), all Serious Adverse Events (SAEs) and communications of the Data Safety Monitoring Board (DSMB). Medicines Development was study Sponsor, as defined in the US Code of Federal Regulations, Title 21, Chapter I, Subchapter D, Subpart D, Part 312.50.

### Study subjects

Subjects were required to be: between 18 and 60 years of age; receiving stable ART for a minimum of 2 months prior to baseline (Day 0), undetectable (<50 copies/mL) plasma viral load for 6 months prior to baseline; CD4 T-cell counts >350 cells/mL at screening with a nadir >100 cells/mL and a positive *ex vivo* or 10 day cultured IFNγ ELIspot assay to OPAL-HIV-Gag(c) peptides. Patients were excluded for receipt of immunomodulatory agents/vaccine 60 days prior to screening or any blood products within 6 months prior to screening. The full entry criteria are available at www.clinicaltrials.gov NCT01123915. All patients provided written informed consent.

### OPAL-HIV-1001 vaccine preparation and administration

**Figure 1:**
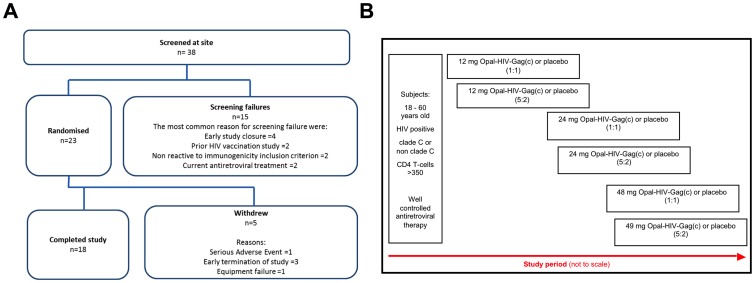
Study subject disposition and allocation to dosing cohorts. (A): Thirty eight subjects were screened for the study, with 23 randomised and 18 completing the study. (B): Diagram showing the planned study allocation to dose escalating cohorts (5∶2) and with sentinel cohorts (1∶1) shown for the 12 mg, 24 mg and 48 mg dose groups.

Subjects were randomised and sequentially allocated to 12 mg, 24 mg or 48 mg OPAL-HIV-Gag(c) or matching placebo in a ratio of 2∶1 (6 active and 3 placebo recipients) (Fig. 1). Subjects were stratified by clade (C or non-C) of HIV infection.

OPAL-HIV-Gag(c) was comprised of 120 15mer peptides spanning the Durban consensus sequence of Clade C Gag, overlapping by 11 amino acids (see below). The peptides were manufactured according to the current Good Manufacturing Practice (cGMP) as defined by United States 21 Code of Federal Regulations by CS Bio Co. (Menlo Park, CA, United States, US). For administration, OPAL-HIV-Gag(c) was reconstituted in 4% volume/volume DMSO Eu Pharm/USP and water for injection (USP). Placebo was DMSO Eu Pharm/USP only (4% volume/volume). Human PBMCs were unaffected by this concentration of DMSO *in vitro* [32].

Blinded study vaccine or matching placebo was administered by drawing 120 mL of whole blood and enriched *ex vivo* for white blood cells by centrifugation using a closed system cell preparation device (Sepax S-100, Biotest). This method generated a range of 253 to 712 million white blood cells concentrated in a 20 mL volume. After enrichment, 12 mg, 24 mg or 48 mg of OPAL-HIV-Gag(c) or placebo, equivalent to 0.6 mg/ml, 1.2 mg/ml, 2.4 mg/ml or 0 mg/ml, respectively, was incubated with the enriched white blood cells for 1 hour at 37°C prior to intravenous reinfusion without wash. The lowest dose equated to the molar exposure shown to be efficacious in non-human primates. The peptide pulsing and cell separation process resulted in median 32.7 µg and 50.3 µg of total peptide per million white blood cells, which is equivalent to 0.27 µg and 0.42 µg for each of the 120 15mer overlapping peptides per million white blood cells for the 12 mg and 24 mg dose groups, respectively.

Study subjects received vaccine administrations at Weeks 0, 4, 8 and 12. Peripheral blood for immunogenicity testing was drawn at Weeks 0, 10, 12, 13, 14 and 16.

### Lymphocyte, CD4 counts, HLA typing and viral load measurement

Lymphocyte count, CD4 count and viral load were measured throughout the study. HLA was typed as previously described [5].

### Peptides

For functional immunogenicity assays, four different HIV specific peptide pools were used (Gag, Rev, Nef and Tat) and one CMV specific peptide pool consisting of 15 amino acids long and overlapping by 11 amino acids spanning the CMV pp65 protein. The Gag peptides were identical to OPAL-HIV-Gag(c). Rev, Nef, and Tat peptides were obtained from the AIDS reagent repository, USA and CMV peptides were obtained from the International AIDS Vaccine Inititative (IAVI) and used for functional assays only.

### *Ex vivo* and cultured interferon gamma ELIspots

We used a previously validated IFNγ ELIspot assay [33] to determine peptide specific responses before and after vaccination. Briefly, for the *ex vivo* ELIspot stimulations, peptide pools at 1,5 µg/peptide/mL or no peptide (cell media only) control was used for 16 to 24 hours stimulation of 200,000 freshly isolated PBMCs per well. The number of specific spot forming units (SFU) was calculated by subtracting the mean number of spots counted in the no peptide control wells from the number of spots counted in each peptide stimulated well performed in triplicates. For the cultured ELIspot assay, 1–2 million PBMCs were added to a 24 well plate in a total volume of 1 mL R10 supplemented with 25 ng of recombinant human (rh) interleukin (IL)-7 (R&D). Cells were stimulated by adding 1.5 µg/mL of each peptide from the OPAL-HIV-Gag(c) pool or media alone at Day 0. By Day 3 all wells were supplemented with 100 units rhIL-2 (Roche) per mL. On Day 7, culture media were replenished by removing 0.5 mL and replacing with 1.5 mL fresh R10/IL-2. On Day 10, cells were recovered to falcon tubes, washed twice in R10 and rested in 1 mL R10 for 24 to 30 hours in 37°C humidified in 5% CO_2_. On Day 11, ‘OPAL-HIV-Gag(c)’ and ‘no peptide’ line were used in the validated IFNγ ELIspot assay as described for the *ex vivo* ELIspot assay. We used 100,000 cells in each well and reported the number of SFU per million cells. Based on OPAL-HIV-Gag(c) stimulation using a sample size of n = 34 HIV-negative individuals (data not shown), the cut-off for a positive OPAL-HIV-Gag(c) response was determined to be >20 SFU (mean 2.4 SFU, <9.97 SFU 99.9% CI) and >300 SFU (mean 76 SFU, <300 SFU 0.88–0.99% CI) per million PBMCs, for the *ex vivo* and cultured IFNγ ELIspot assays, respectively. Viable counting of all PBMC and peptide lines cells were standardised by use of an automated cell counter (Vi-cell XR, Beckman Coulter) to standardise cell counts. We used an AID ELIspot reader to count the number of SFU in each well and presented the data by GraphPad Prism version 5.0d.

### Intracellular cytokine staining

We used intracellular cytokine staining (ICS) assay to detect peptide specific CD4+ and CD8+ T-cell responses before and after vaccination at Week 0, 13 and 14 from primary PBMCs processed from frozen. The principles of this assay are previously described [34]. Briefly, frozen PBMCs were thawed and rested overnight and stimulated with either no peptide (cell media, R10), Gag, CMVpp65 peptide pools (2 µg/peptide/mL) or positive control stimulation (staphylococcal enteroxin B, SEB) (1 µg/mL) (Sigma) in the presence of anti-human CD107a-PE-Cy5 (75 µl/mL) (BD) degranulation marker costimulatory antibodies CD49d, CD28 (1 µg/mL) (BD) and brefeldin A (10 µg/mL) (Sigma) for 6 hours at 37°C in a 5% CO_2_ incubator and stored overnight in 5°C. All stimulations were performed in triplicates except SEB stimulation. The day after stimulation, cells were surface stained with live/dead cell marker (Invitrogen), anti-human CD3-PacificOrange (Invitrogen), CD4-Qdot605 (Invitrogen), CD8-PacificBlue (BD), CD45RA-AlexaFlour700 (BD), CCR7-PE (R&D) then fixed and permeabilised using cytofix/cytoperm kit (BD) and stained intracellularly with anti-human IFNγ-PE-Cy7, IL-2-APC and MIP1β-FITC (R&D) and fixed in 2% paraformaldehyde. All antibodies were pre-titrated before use. Cells were acquired on a LSRII flow cytometer within 24 hours post staining. FlowJo version 8.8.2 was used for data analysis with the following gating strategy; singlets→lymphocytes→live cells→CD3^+^→either CD4^+^ or CD8^+^→IFNγ^+^/MIP1β+ double positive cells to ensure low background. Double positive IFNγ/MIP1β+ values were depicted using GraphPad prism version 5.0a.

### Statistical analysis

The Mann-Whitney U test was used to compare median values for immunogenicity testing between different weeks for both IFNγ ELIspot and ICS assays and for comparing percentage change of lymphocyte counts to Baseline. The Spearman rank correlation coefficient was determined to test correlation of IFNγ ELIspot to ICS assays.

## Results

### Characterisation of recruited individuals for OPAL-HIV-Gag(c) vaccination

Overall, 38 subjects were screened and 23 satisfied the inclusion and exclusion criteria and were randomised to receive 12 mg (n = 6), 24 mg (n = 7), 48 mg (n = 2) or placebo (n = 8) (Fig. 1). Five subjects withdrew from the study: 1 due to equipment failure prior to treatment administration (this patient was replaced); 1 receiving 48 mg withdrew due to a serious adverse event (SAE) leading to early study termination and three subjects (n = 1 48 mg, n = 2 placebo) were required to withdraw when the study was terminated (see Jackson, A. et al PlosOne 2013). In addition, one subject with elevated ALT due to concurrent therapy withdrew from treatment but remained on the study. Because the adverse event occurred in the first subject to receive 48 mg, and led to study discontinuation, there were no subjects in the 48 mg cohort available for immunological assessment. Of the placebo, 12 mg or 24 mg OPAL-HIV-Gag(c) cohorts, the median CD4 T-cell counts were 476, 453 and 654 per µL, respectively, and HAART suppressed plasma viral loads <20 HIV RNA copies/mL at Baseline (Table 1). Each cohort had one individual expressing the protective allele HLA-B*57 and the 24 mg cohort also included one individual expressing the protective allele HLA-B*27:05. All three cohorts represented HLA-B alleles known to restrict at least one or more Gag epitopes. Thus, the three cohorts completing the trial exhibited similar characteristics of protective HLA alleles, CD4+ T-cell counts, treatment suppressed viral load, HIV clade, age and sex distribution.

**Table 1:** HIV-1 seropositive subjects under HAART treatment used for ‘Opal-HIV-Gag(c)’ vaccine administration.

HIV-1 seropositive subjects under HAART treatment used for ‘Opal-HIV-Gag(c)’ vaccine administration													
		HLA class I											
Subject ID	Dose	A1	A2	B1	B2	Cw1	Cw2	CD4 [cells/µL]^a^	HIV RNA [copies/mL]^a^	HAART	HIV Clade	Age [yrs]^a^	Sex
002	0 mg	3001	3104	4201	4501	0602	1701	362	<20	yes	C	42	F
003		0201	3601	1503	5301	0210	0401	904	<20	yes	C	47	F
109		0201	0201	nd	nd	0303	0702	469	<20	yes	B	46	M
011		0101	0302	3501	5701	0602	0602	1421	<20	yes	C	56	M
013		2902	3001	0702	0801	0702	0702	406	<20	yes	C	47	M
018		0101	2601	0702	5201	0702	1202	483	<20	yes	B	39	M
median								476	<20			47	
001	12 mg	0201	3402	3501	3910	1203	1601	433	<20	yes	C	43	M
004		0201	2301	0702	4901	0701	0702	480	<20	yes	C	56	F
005		0205	0301	1402	5001	0602	0802	370	<20	yes	C	32	F
006		2902	3201	1302	4403	0602	1601	760	<20	yes	B	44	M
007		0201	0301	1402	5703	0802	0802	395	<20	yes	A1	45	M
008		3301	6601	1402	5301	0401	0802	472	900	yes	BF	37	M
median								453	<20			44	
010	24 mg	0201	0201	1301	1501	0102	0304	696	<20	yes	C	28	M
012		0202	2902	4901	5703	0701	0701	611	<20	yes	C	34	F
014^b^		3402	3601	4403	5301	0401	0401	556	<20	yes	C	36	F
015		0201	0205	0705	4901	0701	0701	740	<20	yes	B	41	M
016		0101	2402	3508	5201	0401	1202	1074	<20	yes	B	33	M
017		0201	0201	1501	2705	0202	0303	361	<20	yes	B	43	M
median								654	<20			35	
019^b^	48 mg	0201	0201	1801	3501	0401	0701	859	<20	yes	C	46	M
024^b^		0101	0301	4101	4901	0602	0701	481	<20	yes	B/D	54	M
median								670	<20			50	

a Values obtained at baseline defined as day of first vaccination.

b Withdrawn from study after two administrations (014) or after one administration (019 and 024).

### Pre-existing OPAL-HIV-Gag(c) specific responses could be boosted *in vitro*

**Figure 2:**
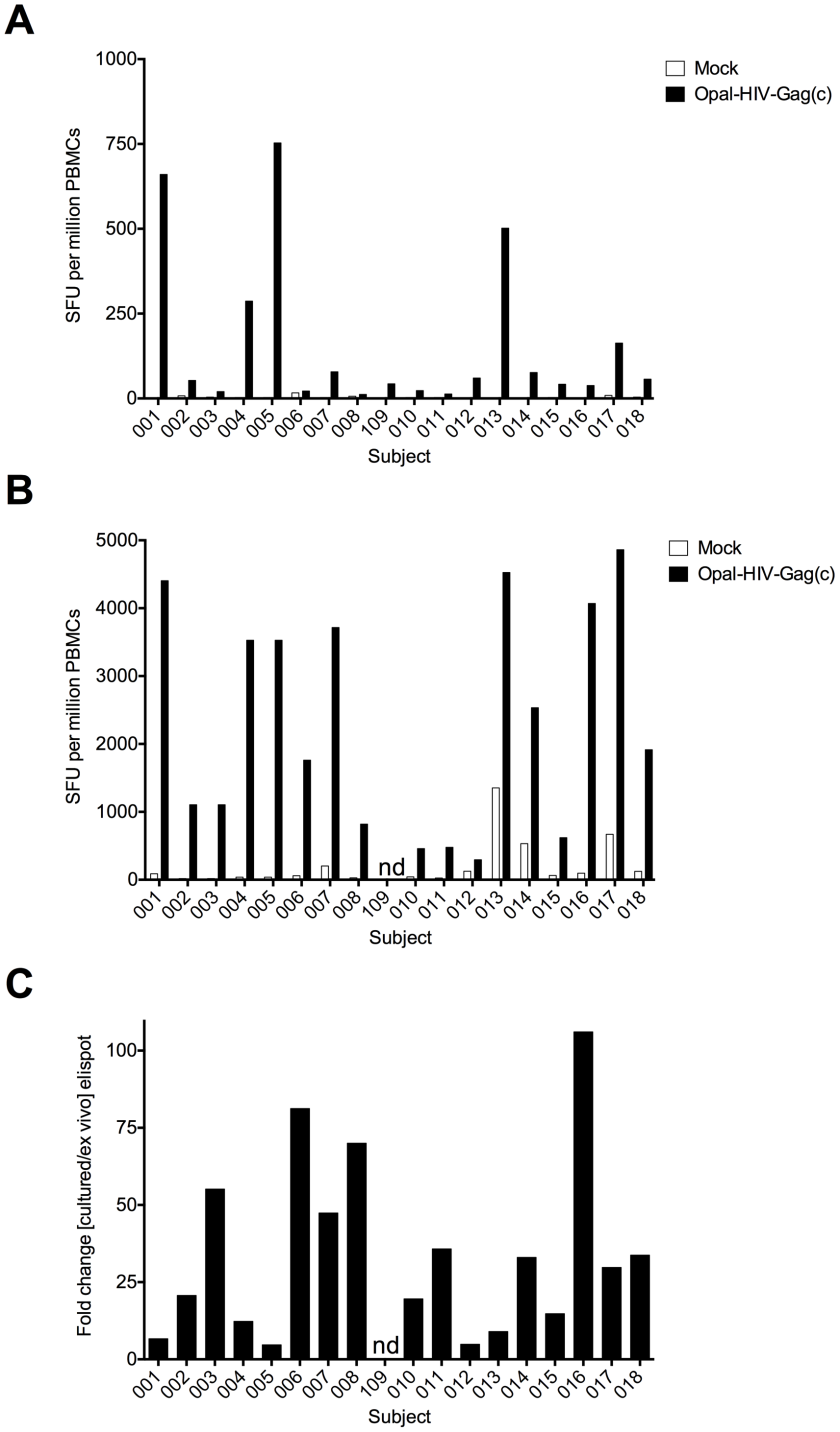
Magnitude and expansion potential of pre-existing OPAL-HIV-Gag(c) specific responses. Eighteen subjects completing the study were tested for IFNγ ELIspot responses expressed as SFU per million inpuT-cells to OPAL-HIV-Gag(c) peptides or mock (media only) from fresh ex vivo PBMCs (A) or from 10 day cultured OPAL-HIV-Gag(c) peptide expanded PBMCs (B) from screening samples available at 2–6 weeks prior to baseline. The expansion capacity was determined as the fold change of magnitude for the cultured ELIspot over the ex vivo ELIspot (C). ND not done.

All of the 18 enrolled subjects showed pre-existing Gag specific responses, measured either *ex vivo* or by 10 day cultured IFNγ ELIspot, 2 to 6 weeks before Baseline with median OPAL-HIV-Gag(c) specific responses of 55 (range 12–753) and 2335 (range 457–4523) SFU/million PBMCs, respectively (Fig. 2A and B). We detected low-frequency *ex vivo* responses to Rev, Nef and Tat (median 8, 55, 5 SFU/million PBMCs, respectively) (Fig. S1) and the expected high-frequency CMV-specific responses (median 1518, range 233–3098 SFU/million PBMCs) (data not shown). To test whether the pre-existing Gag specific response from each of the enrolled individuals found in the *ex vivo* ELIspot assay had the potential to be boosted *in vitro*, we compared the *ex vivo* Gag-specific responses to the 10 day *in vitro* expanded responses and found each of the responses was significantly boosted (median fold increase 30, range 5–106-fold increase) (Fig. 2C). Thus, all of the enrolled subjects had detectable pre-existing OPAL-HIV-Gag(c) specific responses, which could be boosted *in vitro*, suggesting proliferative functional capabilities for *in vivo* boosting with OPAL-HIV-Gag(c).

### Limited boosting of Gag specific CD8+ T-cell responses after OPAL-HIV-Gag(c) vaccination

**Figure 3:**
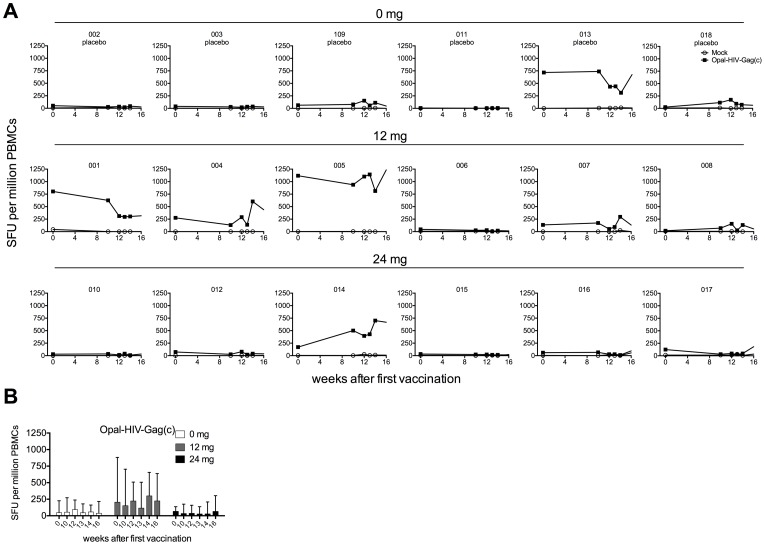
OPAL-HIV-Gag(c) peptide pool specific responses before and after vaccination. All six subjects from each dose group (0 mg, 12 mg and 24 mg) were tested for OPAL-HIV-Gag(c) specific or no peptide (mock) responses by IFNγ ex vivo ELIspot performed from fresh cells at week 0, 10, 12, 13, 14 and 16 after first vaccination expressed as the mean SFU per million cells of triplicate stimulations (A) and expressed as median values within dose groups with error bars representing inter quartile ranges (B).

No boost of Gag-specific responses was observed following vaccination in the placebo or in the 12 mg or 24 mg dose cohorts, comparing median SFU/million PBMCS at baseline with Week 13, Week 14 or Week 16 for placebo, 12 and 24 mg dose cohorts (45 vs 38, 206 vs 224, 67 vs 66 SFU/million PBMCS, respectively) (Fig. 3). Individual results were variable. For two subjects in the 12 mg dose group, responses in subject 001 decreased from 803 to 317 SFU/million PBMCs and in subject 004 responses increased from 277 at Baseline to 438 SFU/million PBMCs at Week 16; and in one subject in the 24 mg dose group, responses in Subject 014 increased from Baseline of 171 to 668 SFU/million PBMCs at Week 16. However, Subject 014 only received two doses (Week 0 and 4) due to an increase in ALT (see Jackson et al, 2013). When we examined antigen specific responses to Rev, Nef, Tat or CMV, which were not contained in the OPAL-HIV-Gag(c) vaccine, we did not detect any change in magnitude of responses in any of the groups. (Fig. S2). In conclusion, the OPAL-HIV-Gag(c) administrations did not have a significant effect on Gag specific responses as measured by the IFNγ ELIspot assay, despite 6 of the 12 subjects in the active groups having pre-existing Gag responses of more than 125 SFU/million PBMCs.

**Figure 4:**
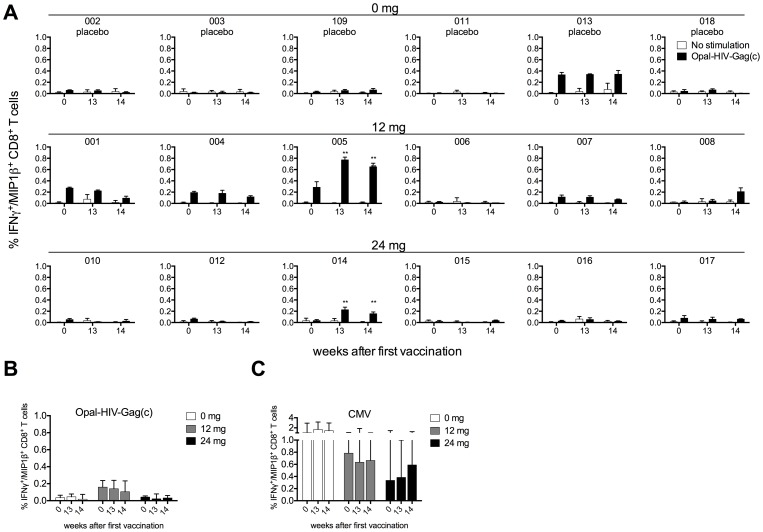
OPAL-HIV-Gag(c) and CMV peptide pool specific CD8+ T-cell responses before and after vaccination. All six subjects from each dose group (0 mg, 12 mg and 24 mg) were tested for OPAL-HIV-Gag(c) specific or no peptide (no stimulation) responses by ICS shown as IFNγ+/MIP1β+ double positive CD8+ T-cells processed from frozen PBMCs derived at week 0, 13 and 14 after first vaccination expressed as the mean of triplicate stimulations (A) and expressed as median values within dose groups with error bars representing inter quartile ranges for OPAL-HIV-Gag(c) (B) and for CMV specific CD8+ T-cell responses (C).

When we applied an intracellular cytokine staining (ICS) assay to detect IFNγ^+^/MIP1β^+^ producing antigen specific CD8+ T-cell responses at Week 0, 13 and 14, we found similar intersubject patterns of CD8+ Gag-specific responses as observed for the IFNγ ELIspot assay (Fig. 4A and Fig. S3A) with a strong correlation between these two assays (Spearman R = 0.84, P<0.0001, data not shown), indicating that most of the responses detected by IFNγ ELIspot are derived from CD8+ T-cells. Specifically, we detected a small increase in Gag specific CD8+ T-cell magnitudes in subject 005 (0.3% vs 0.8% at Week 0 vs Week 13, respectively) dominated by triple positive IFNγ+/MIP1β+/CD107a+ producing cells, but with no IL-2 production (Fig. S3B), and with no change in memory cell subsets (CCR7 and CD45RA) during vaccination (data not shown). Subject 008 and 014 also showed small increases in magnitude comparing Week 0 and Week 14, whereas 001 had a decreased response. Overall and consistent with the ELIspot data, we did not see any change of Gag-specific responses in the placebo or in the two treatment groups, median % IFNγ^+^/MIP1β^+^ CD8+ T-cells at baseline vs week 14 for placebo, 12 and 24 mg dose groups being 0.03 vs 0.02, 0.16 vs 0.11, 0.04 vs 0.03, respectively (not significant) (Fig. 4). We detected CMV specific CD8+ T-cell responses (>0.1%) in all individuals except subject 008 (Fig. S4) and did not observe any overall change in magnitudes of CMV specific CD8+ T-cell responses at week 0, 13 and 14 (Fig. 4C) consistent with the CMV specific data obtained from the IFNγ ELIspot assays (Fig. S2). All of the subjects responded to the positive control SEB (data not shown). Thus, we did not see any overall boost of OPAL-HIV-Gag(c) specific CD8+ T-cells after vaccination.

### Lack of OPAL-HIV-Gag(c) specific CD4+ T-cell responses

**Figure 5:**
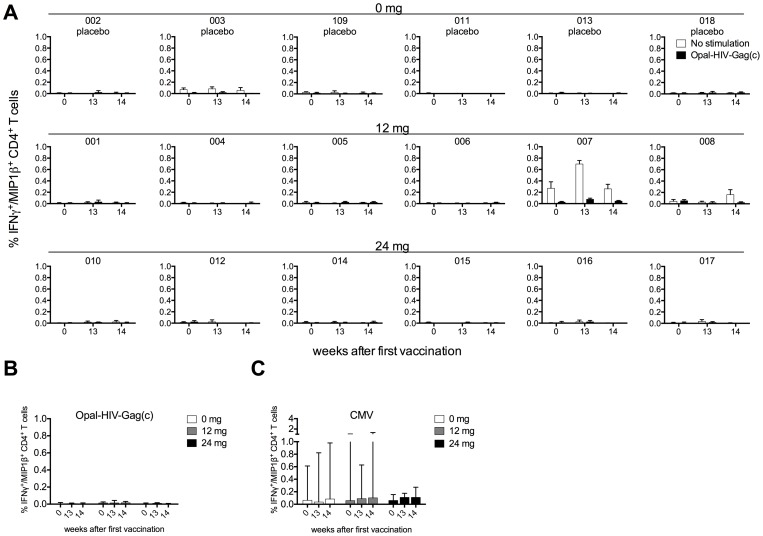
OPAL-HIV-Gag(c) and CMV peptide pool specific CD4+ T-cell responses before and after vaccination. All six subjects from each dose group (0 mg, 12 mg and 24 mg) were tested for OPAL-HIV-Gag(c) specific or no peptide (mock) responses by ICS shown as IFNγ+/MIP1β+ double positive CD4+ T-cells processed from frozen PBMCs derived at week 0, 13 and 14 after first vaccination expressed as the mean of triplicate stimulations (A) and expressed as median values within dose groups with error bars representing inter quartile ranges for OPAL-HIV-Gag(c) (B) and for CMV specific CD4+ T-cell responses (C).

The use of 15 amino acid long peptides had previously boosted CD4+ T-cell responses in the macaque model [29], [30], [31], [35]. Therefore, we undertook ICS assays to determine CD4+ T-cell responses at week 0, 13 and 14 for the three dose cohorts (Fig. 5). We did not detect any CD4+ T-cell responses specific for the vaccine peptides OPAL-HIV-Gag(c) (Fig. 5A and B), but detectable CMV specific CD4+ T-cell responses (>0.1%) were found for 10 out of the 18 subjects (Fig. S5) with no overall change in magnitude of CMV specific CD4+ T-cell responses over the three time points measured (Fig. 5C). Seventeen out of 18 subjects responded (>0.1%) to the positive control SEB (data not shown). Thus, the OPAL-HIV-Gag(c) vaccinations did not boost or induce Gag specific CD4+ T-cell responses *in vivo*.

### Transient lymphopenia immediately after OPAL-HIV-Gag(c) vaccination

**Figure 6:**
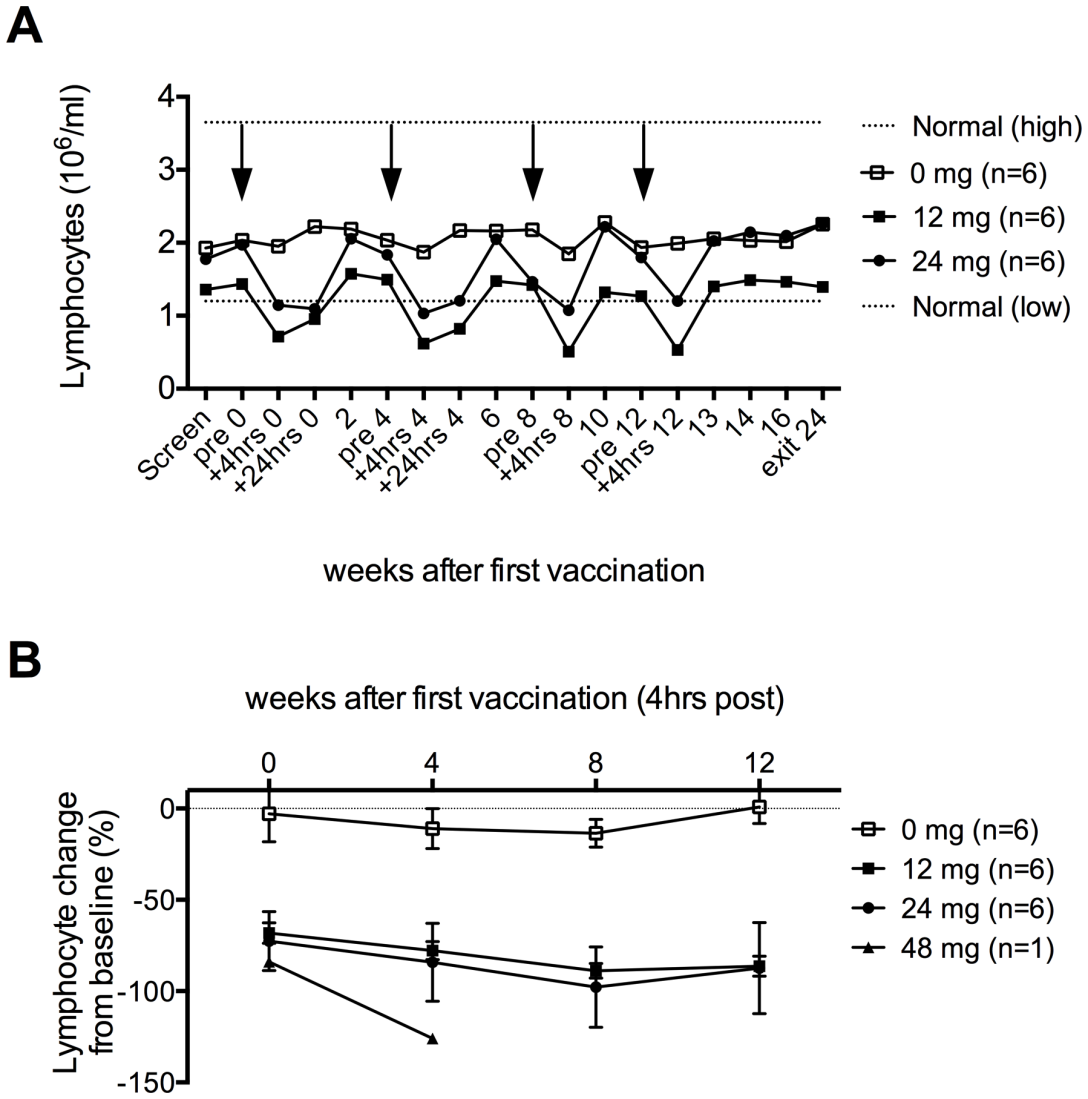
Transient and treatment specific lymphopenia after vaccination. Total lymphocyte counts were performed before, during and at follow up after vaccination as indicated on the x-axis for the three groups (0 mg, 12 mg and 24 mg) and shown as mean values (million lymphocytes per ml whole blood) for the 6 subjects within each group with normal high and low values for HIV positive individuals indicated by dotted lines (A). Arrows indicate vaccinations. The percent (%) change of lymphocyte count from baseline (week 0) is shown as mean values for the three dose groups with error bars representing standard error of mean (SEM) (B). Only one subject (024) was available for the 48 mg dose group at week 0 and 4.

We measured the lymphocyte count at: screening, pre-vaccination, post-vaccination (4 hours after vaccination), discharge (24 hours after vaccination), during follow up weeks 2, 6, 10, 13, 14, 16 and at study exit week 24 for the placebo and OPAL-HIV-Gag(c) dose groups (Fig. 6A). No change was observed within the placebo group (PBMCs pulsed with placebo containing DMSO but no peptide) median 1.7-1.56 million lymphocytes per ml (P = 0.16) (−9% change from Baseline), but a significant OPAL-HIV-Gag(c)-induced reduction of lymphocytes was observed 4 hours post each vaccination (median 1.72 down to 0.67 million lymphocytes per ml (P<0.0001) (−80% change from baseline) (Fig. 6B)). The lymphocytes started re-emerging to pre-vaccination levels between 4 and 24 hours post-vaccination and fully resolved within 2 weeks post vaccination (measurements were not made between 24 hrs post-vaccination and 2 weeks afterwards). The rapid rebound of lymphocytes suggests that the lymphocytes are not depleted, but temporarily migrated out of the peripheral blood and subsequently re-emerge within 2 weeks.

## Discussion

The OPAL method was shown to be highly immunogenic and effective in reducing viral load in SIV infected pigtail macaques [30], [31], [35] and, thus, an attractive candidate for testing in humans. However, OPAL-HIV-Gag(c) treatment in the clinic showed no boosting effect on Gag-specific CD8+ or CD4+ T-cells. Subjects were able to mount an *in vitro* response to OPAL peptides and a biological effect was observed: subjects receiving OPAL-HIV-Gag(c) but not placebo exhibited a transient, self-limiting lymphopenia immediately post-dose. This study was prematurely terminated due to a single serious adverse event and a cause other than the study product could not be identified, as described elsewhere (see Jackson A. et al. PlosOne 2013).

This Phase I, first-in-human study had a primary endpoint of safety with a secondary endpoint to assess immunogenicity. Therefore, the study population used here was chosen specifically to the primary endpoint rather than a stage of HIV infection to mimic the non-human primate studies. Key differences included the timing of vaccination relative to initial infection and the presence or absence of concurrent ART. In the macaque studies, ART control of acute viraemia was induced 3 weeks post infection, thereby preserving a healthy CD4+ T-cell pool [36], including fresh primed SIV specific CD4+ and CD8+ T-cell responses. In contrast, the human volunteers for this study were chronically infected before initiation of ART. By definition, they had met the criteria to start ART because their immune system was compromised, and absolute CD4 counts depleted, although nadir CD4 counts in each case was more than 100 cells/µL. Nonetheless an absolute CD4 count as low as 100 cells/mm^3^ represents relatively severe immunocompromise. In addition, although the *in vitro* expansion of Gag-specific CD8+ T-cells during cultured ELIspot assays (Fig. 2) suggested a capacity for proliferation and augmentation of the Gag-specific response by OPAL-HIV-Gag(c) administration, these assays included IL-2 addition to the culture medium which may not have been present *in vivo*. However, it was possible to boost SIV-specific responses in chronically infected macaques using this approach [35] suggesting that the absence of responses observed in this human trial is not entirely explained by the timing of ART initiation in the human study subjects.

The OPAL-HIV-Gag(c) treatment induced lymphopenia was treatment specific, since it did not occur in the placebo group, was transient, with partial recovery of peripheral lymphocyte counts within 24 hours and full recovery by next measurement (2 weeks). The precise kinetic of recovery of the peripheral lymphocyte numbers are unknown as there were no measurements between 4 hours and 2 weeks post-treatment. However, the indications of rapid recovery suggest redistribution of lymphocytes. One may speculate that changed patterns of cell trafficking resulted via peptide induced expression of homing receptors, such as CCR7, a ligand for CCL21 expressed on high endothelial venules, facilitates altered cell migration, especially on CCR7 positive naïve T-cells [37].

It is necessary to test effective non-human primate vaccines in human clinical trials in search for signals to an effective HIV vaccine. However, this OPAL-HIV-Gag(c) first-in-man study did not replicate the high T-cell immunogenicity observed in the promising pigtail macaque studies [30], [31], [35] and further dosing was limited by a serious adverse event. Inconsistency between pre-clinical non-human primate and human clinical trials has been frequently reported [10], [11], [12], [13], [38], [39]. The failure of the HIV efficacy STEP trial [15] arose despite prior efficacy observed from non-human primate studies [14], [40], [41], [42] and immunogenicity in HIV negative individuals [43], [44]. Although this current trial was intended to evaluate the safety as a primary endpoint, we conclude that the lack of immunogenicity (secondary endpoint) observed here warrants emphasis on alternative vehicle delivery systems for the HIV Gag immunogen.

## Supporting Information

Figure S1 **Magnitude of pre-existing HIV Rev, Nef and Tat specific responses.** Eighteen subjects completing the study were tested for IFNγ ELIspot responses expressed as SFU per million inpuT-cells to HIV peptide pools Rev, Nef, Tat or mock (media only) from fresh ex vivo PBMCs obtained from screening samples available at 2–6 weeks prior to baseline.
(TIFF)

Figure S2 **HIV Rev, Nef, Tat and CMV peptide pool specific responses before and after vaccination.** All six subjects from each dose group (0 mg, 12 mg and 24 mg) were tested for HIV Rev, Nef, Tat and CMV peptide specific responses or no peptide by IFNγ ex vivo ELIspot performed from fresh cells at week 0, 10, 12, 13, 14 and 16 after first vaccination expressed as median values within dose groups with error bars representing inter quartile ranges.
(TIFF)

Figure S3 FACS plots showing CD8+ T-cell gating strategy (top panel) with effecter producing CD8+ T-cells shown after no stimulation, OPAL-HIV-Gag(c) or SEB stimulation at week 13 for subject 005 (A) and shown as boolean gated polyfunctional pie charts examining CD107a/IFNγ/IL2/MIP1β producing total CD8+ T-cells at 3 time points for subject 005 (B).
(TIFF)

Figure S4 **Subject individual CMV peptide pool specific CD8+ T-cell responses before and after vaccination.** All six subjects from each dose group (0 mg, 12 mg and 24 mg) were tested for CMV specific responses by ICS shown as IFNγ+/MIP1β+ double positive CD8+ T-cells processed from frozen PBMCs derived at week 0, 13 and 14 after first vaccination expressed as the mean of triplicate stimulations and shown for each individual.
(TIFF)

Figure S5 FACS plots showing CD4+ T-cell gating strategy (top panel) with effecter producing CD4+ T-cells shown after no stimulation, OPAL-HIV-Gag(c) or SEB stimulation at week 13 for subject 005 (A) and in (B) All six subjects from each dose group (0 mg, 12 mg and 24 mg) were tested for CMV specific responses by ICS shown as IFNγ+/MIP1β+ double positive CD4+ T-cells processed from frozen PBMCs derived at week 0, 13 and 14 after first vaccination expressed as the mean of triplicate stimulations and shown for each individual.
(TIFF)

Checklist S1 **Consortium checklist.**
(DOCX)

Protocol S1 **Trial protocol.**
(PDF)
