# Supplementary figures and images for: Non-Immunogenicity of Overlapping Gag Peptides Pulsed on Autologous Cells after Vaccination of HIV Infected Individuals
Source: PLoS One. 2013 Oct 4;8(10):e74389. doi: 10.1371/journal.pone.0074389 (PMC3790804; doi:10.1371/journal.pone.0074389)

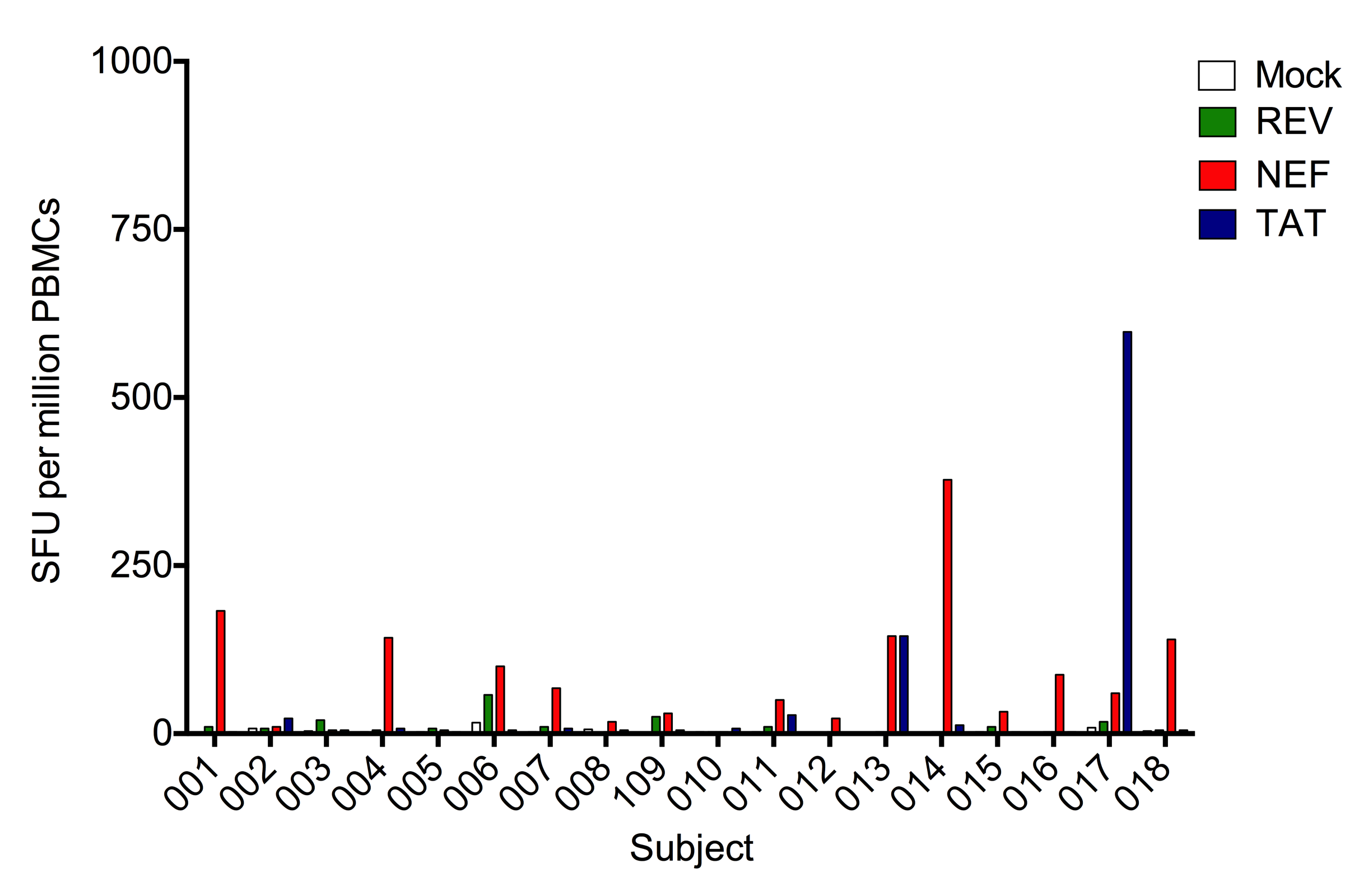

Supplement: Figure S1 — Magnitude of pre-existing HIV Rev, Nef and Tat specific responses. Eighteen subjects completing the study were tested for IFNγ ELIspot responses expressed as SFU per million inpuT-cells to HIV peptide pools Rev, Nef, Tat or mock (media only) from fresh ex vivo PBMCs obtained from screening samples available at 2–6 weeks prior to baseline. (TIFF) [file pone.0074389.s001.tiff]

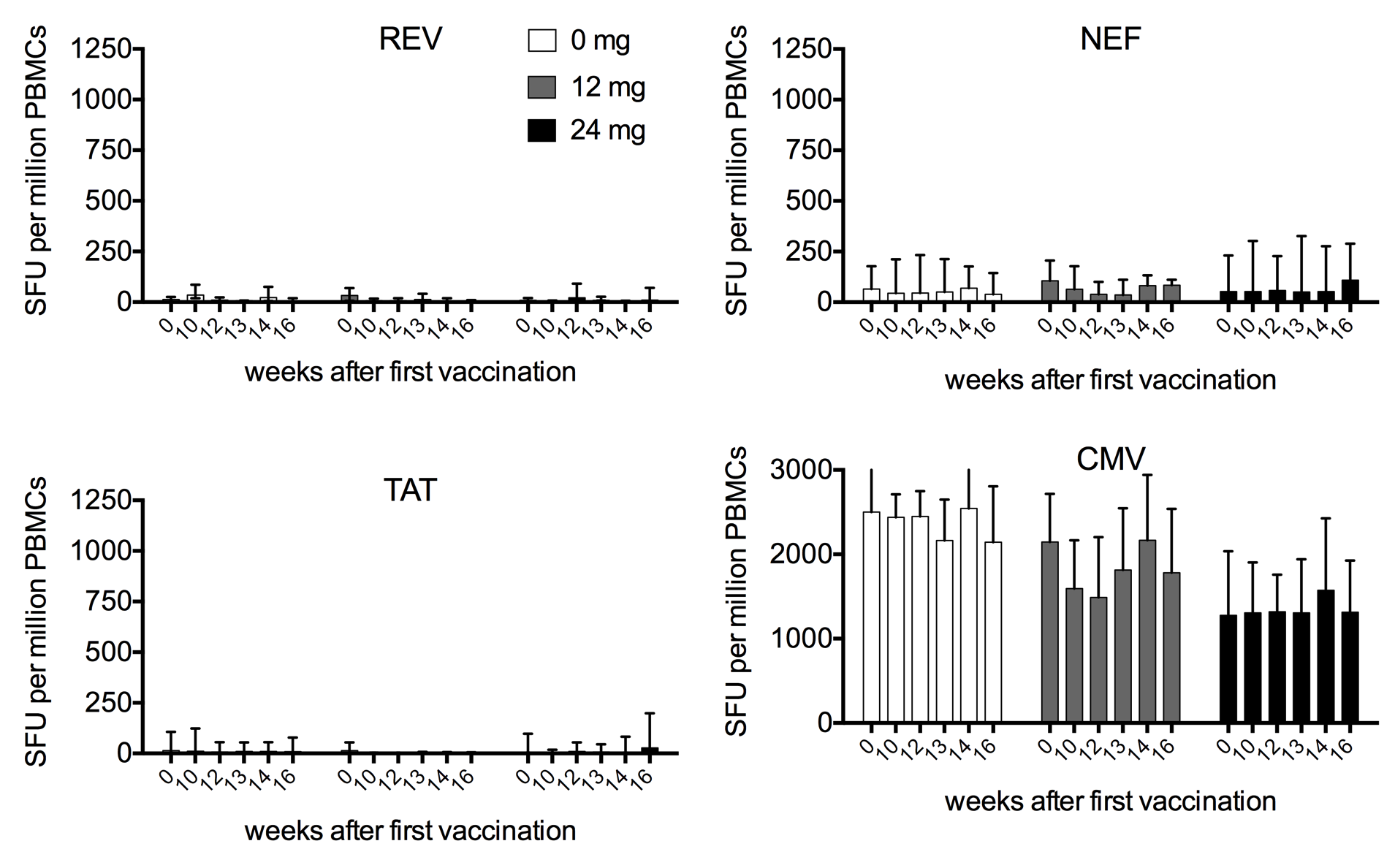

Supplement: Figure S2 — HIV Rev, Nef, Tat and CMV peptide pool specific responses before and after vaccination. All six subjects from each dose group (0 mg, 12 mg and 24 mg) were tested for HIV Rev, Nef, Tat and CMV peptide specific responses or no peptide by IFNγ ex vivo ELIspot performed from fresh cells at week 0, 10, 12, 13, 14 and 16 after first vaccination expressed as median values within dose groups with error bars representing inter quartile ranges. (TIFF) [file pone.0074389.s002.tiff]

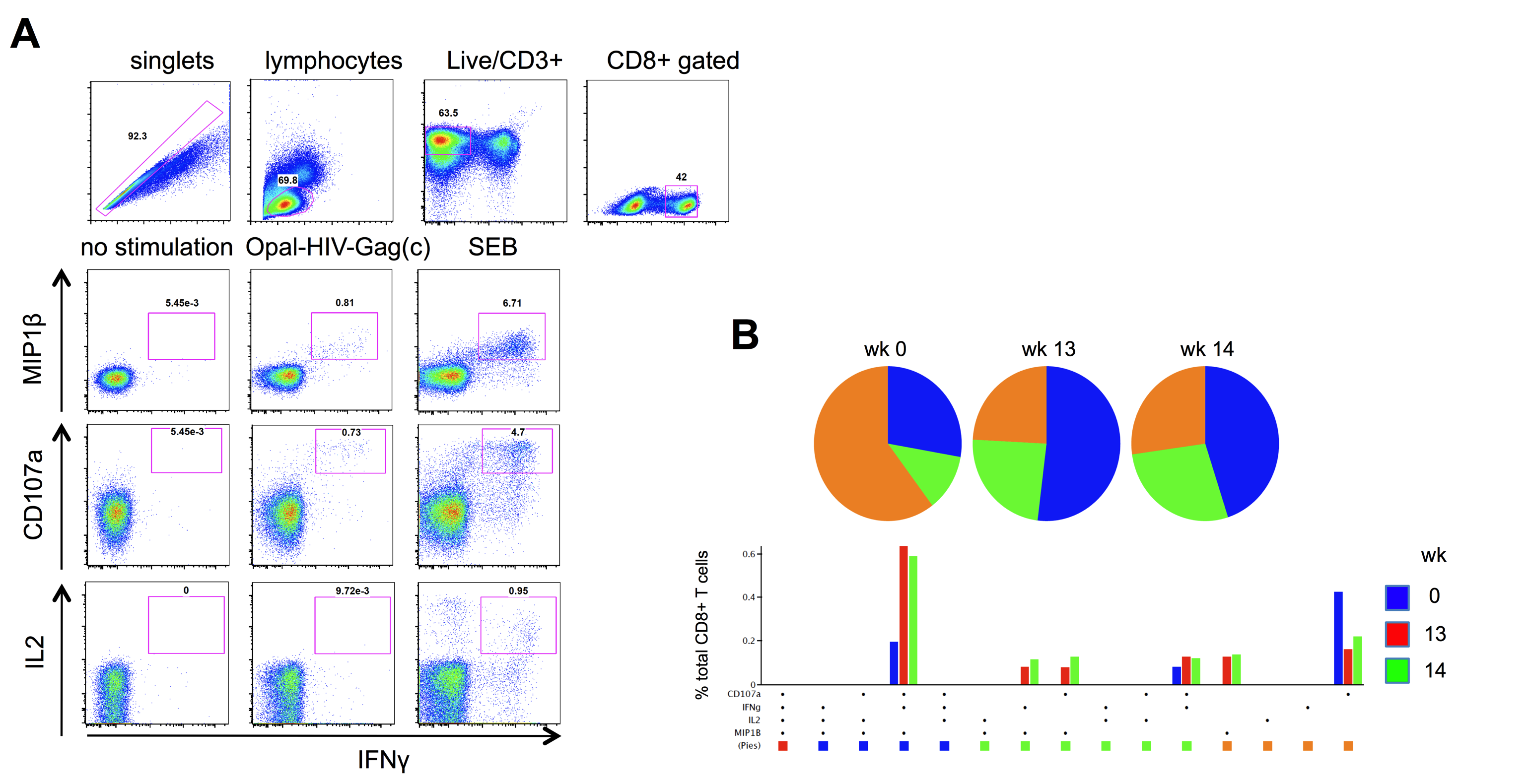

Supplement: Figure S3 — FACS plots showing CD8+ T-cell gating strategy (top panel) with effecter producing CD8+ T-cells shown after no stimulation, OPAL-HIV-Gag(c) or SEB stimulation at week 13 for subject 005 (A) and shown as boolean gated polyfunctional pie charts examining CD107a/IFNγ/IL2/MIP1β producing total CD8+ T-cells at 3 time points for subject 005 (B). (TIFF) [file pone.0074389.s003.tiff]

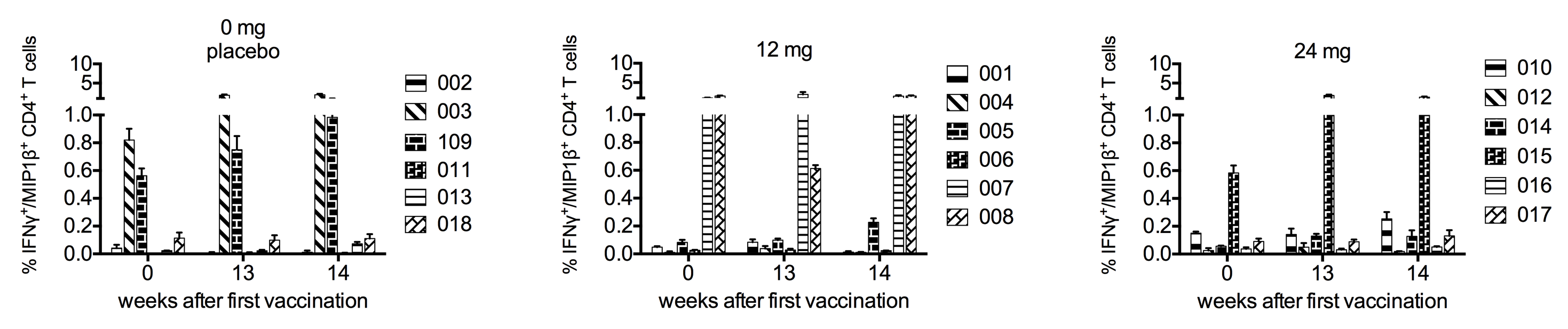

Supplement: Figure S4 — Subject individual CMV peptide pool specific CD8+ T-cell responses before and after vaccination. All six subjects from each dose group (0 mg, 12 mg and 24 mg) were tested for CMV specific responses by ICS shown as IFNγ+/MIP1β+ double positive CD8+ T-cells processed from frozen PBMCs derived at week 0, 13 and 14 after first vaccination expressed as the mean of triplicate stimulations and shown for each individual. (TIFF) [file pone.0074389.s004.tiff]

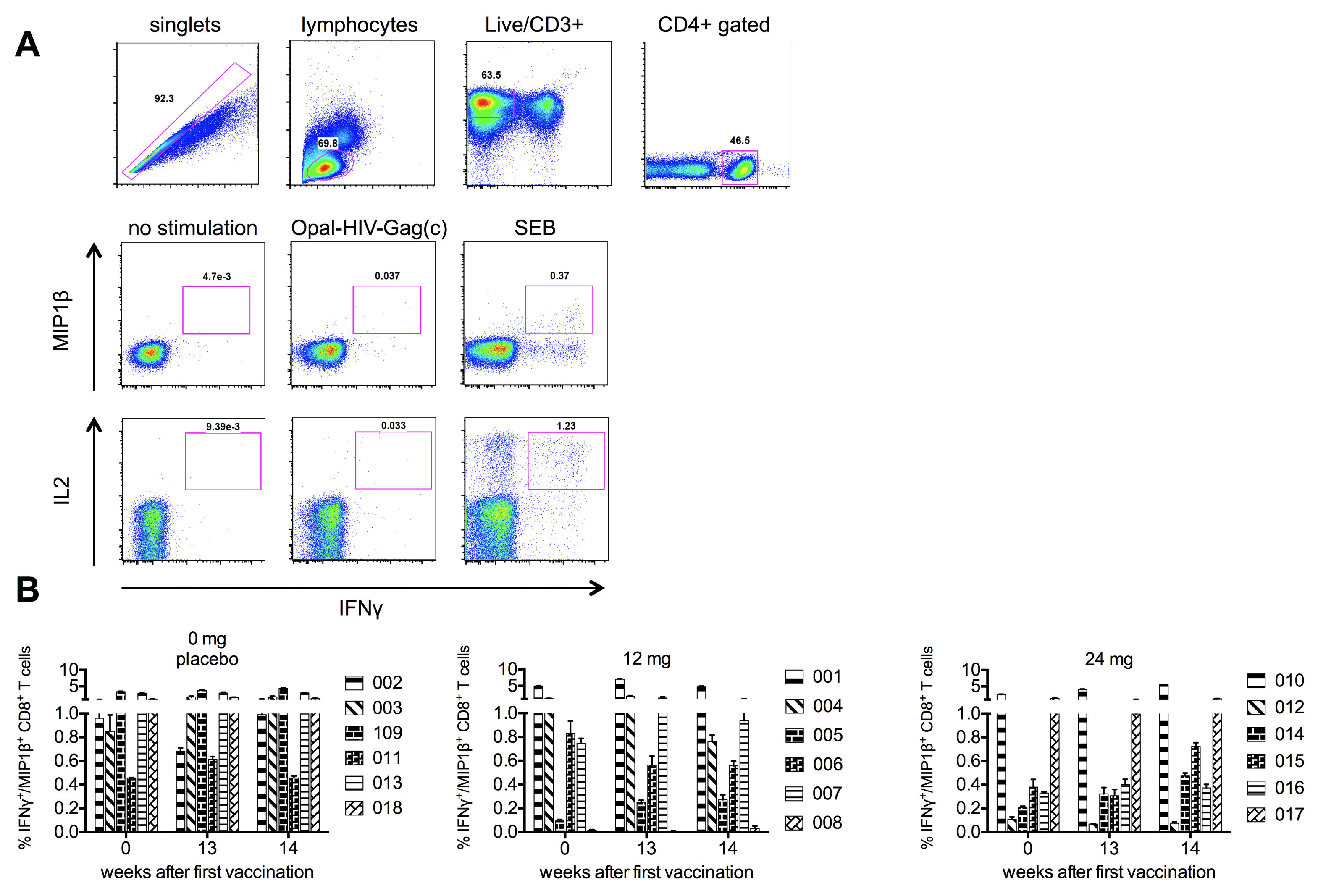

Supplement: Figure S5 — FACS plots showing CD4+ T-cell gating strategy (top panel) with effecter producing CD4+ T-cells shown after no stimulation, OPAL-HIV-Gag(c) or SEB stimulation at week 13 for subject 005 (A) and in (B) All six subjects from each dose group (0 mg, 12 mg and 24 mg) were tested for CMV specific responses by ICS shown as IFNγ+/MIP1β+ double positive CD4+ T-cells processed from frozen PBMCs derived at week 0, 13 and 14 after first vaccination expressed as the mean of triplicate stimulations and shown for each individual. (TIFF) [file pone.0074389.s005.tiff]
